# Outbreak investigation of foot-and-mouth disease in cattle in Tigray region, Northern Ethiopia

**DOI:** 10.3389/fvets.2023.1157395

**Published:** 2023-08-14

**Authors:** Adehanom Baraki Tesfaye, Guash Abay Assefa, Leul Berhe Shishaye, Bisrat Mesfin Abera, Nechey Tsehaye Gebreanenya, Gebru Legesse Gebregiorgis, Salome Dürr

**Affiliations:** ^1^Mekelle Agricultural Research Center, Tigray Agricultural Research Institute, Mekelle, Ethiopia; ^2^Abergelle Agricultural Research Center, Tigray Agricultural Research Institute, Abi Adi, Ethiopia; ^3^Humera Begait Research Center, Tigray Agricultural Research Institute, Humera, Ethiopia; ^4^Tigray Bureau of Agriculture and Rural Development, Animal Health Core-process, Mekelle, Ethiopia; ^5^Veterinary Public Health Institute, University of Bern, Bern, Switzerland

**Keywords:** cattle, FMDV, Kafta Sheraro National Park, outbreak, participatory epidemiology, Western Tigray, wildlife, vaccination

## Abstract

An investigation of a foot-and-mouth disease (FMD) outbreak was conducted between late October and mid-December 2019 in Tigray region. The outbreak investigation team collected epidemiological data from the six villages of Kafta Humera and Seharti Samre districts, including morbidity proportions, mortality proportions, and clinical signs, and cattle management and vaccination history were collected via participatory methods, including interviews and group discussions with local experts and farmers in Kafta Humera and reports from the district veterinarians in Seharti Samre. Twenty-two tissue samples were collected for laboratory confirmation. Overall, 4,299/9,811 (43.8%) and 13,654/16,921 (80.6%) cattle showed clinical signs for FMD in Kafta Humera and Seharti Samre, respectively. In Kafta Humera, the highest morbidity proportion was found in adult cows and heifers (48.1%), followed by 27.8% in oxen and 15.9% in calves. In Seharti Samre, the morbidity proportion was similar in all age groups at ~81%. No death of FMD-suspected cattle was reported throughout the outbreak. The serotype of foot-and-mouth disease virus (FMDV) identified by laboratory analysis differed between the two districts (serotype O in Kafta Humera and serotype A in Seharti Samre). We, therefore, suggest that the outbreaks in the two districts occurred independently from each other. Experts and farmers were interviewed and believed that the outbreak in Kafta Humera was most likely caused by interaction between cattle and wildlife from the surrounding Kafta Sheraro National Park, which share common grazing land. This outbreak investigation showed that FMD can cause devastating cattle morbidity. A regular vaccination program against the identified circulating FMDV serotypes with sufficient coverage is required to avoid future outbreaks.

## Introduction

Foot-and-mouth disease (FMD) is highly contagious for cloven-hooved animals and is caused by foot-and-mouth disease virus (FMDV), a virus under genus *Aphthovirus* and family Picornaviridae. Transmission of the virus occurs through direct contact with infected cattle and infected aerosols and indirect contact via contaminated feed and water (1, 2). Currently, seven immunologically distinct serotypes have been identified, which are O, A, C, Asia 1, and South African territory (SAT) 1–3, as well as multiple subtypes circulating worldwide (3–6). Currently, most of the industrialized countries (such as Europe, Northern America, or Australia) hold the World Organization for Animal Health (WOAH) certified status of FMD-free without vaccination, after regular vaccination strategies were ceased in most European countries in the early 1990s to facilitate international trade. However, FMD is still endemic in Asia and Africa (7, 8). The impact of FMD on livestock industry in endemic countries is considerable, which is caused by direct (such as production losses, fertility problems, change in herd structure, and delayed sale of livestock) and indirect (such as costs related to vaccination, diagnostic tests, and culling of animals) costs (7). In addition, FMD in endemic countries impedes the access to lucrative international markets for animal or animal product trade. The economic loss of FMD for small holding farmers in endemic countries is estimated at $USD 6.5–21 billion worldwide annually (7), although large information gaps exist for accurate estimates (9).

FMD is endemic in Ethiopia's cattle population with emerging serotypes arising in previously unaffected areas (10). Five of the seven FMDV serotypes (O, A, C, SAT 1, and SAT 2) were identified in Ethiopia during the period of 1981–2007 (11). The temporal and spatial distribution of FMD outbreaks in Ethiopia is complex, mainly due to the presence of several FMDV serotypes and susceptible hosts in wildlife that may act as reservoirs (10). The outbreak frequencies reported from 2008 to 2018 are 4.3 times higher than the reports from 1981 to 2007 (10). Since 2007, FMD outbreaks occur almost every year and affect the entire country (10, 12), including Amhara region (13, 14) and Tigray region (15, 16). Regular outbreaks are also reported from Oromia region (Borena pastoral area), where the main route for export of live animals is located (17, 18). The economic loss for the Ethiopian cattle system is remarkable, with an estimated average of $USD 76 per affected herd in the crop-livestock mixed system and $USD 174 in the pastoral production system (18, 19).

In industrialized farming systems in African countries, controlling FMD by mass vaccination has been shown to be possible (20). Other control measures such as fencing and cattle movement restriction at the wildlife-livestock interfaces have been practiced in South African countries (mainly South Africa), although they are challenging to implement, which may be due to the lack of interest by some owners/herders and high-cost requirements (21). However, in eastern Africa, where the farming system is traditional limiting fencing and movement control and the role of wildlife in the epidemiology of FMD is not clear (10), many countries are not able to create FMD-free zones. Effective vaccines exist to control FMD, yet the protection period after vaccination is short (6 month) with weak cross protection between serotypes (22). In Ethiopia, trivalent FMD vaccines composed of serotype A, O, and SAT2 are commonly produced and distributed all over the country (23). Most traditional (small-scale) farmers are not interested in using these vaccines because of its high price/dose [15 Ethiopian birr, which is equal to ~$USD 0.27 (10)]. Therefore, the vaccine is mostly utilized in the urban and peri-urban commercialized dairy farms and feedlots, leading to low vaccination coverage in most villages of the country. As a consequence, Ethiopia does not meet the requirements of WOAH and the World Trade Organization (WTO) for the international trade of live animals and animal products, leading to the missed opportunity for lucrative international markets (10, 20, 24, 25), though the country owned the largest cattle population in Africa in 2020, nearly 70 million heads (26).

**Figure 1:**
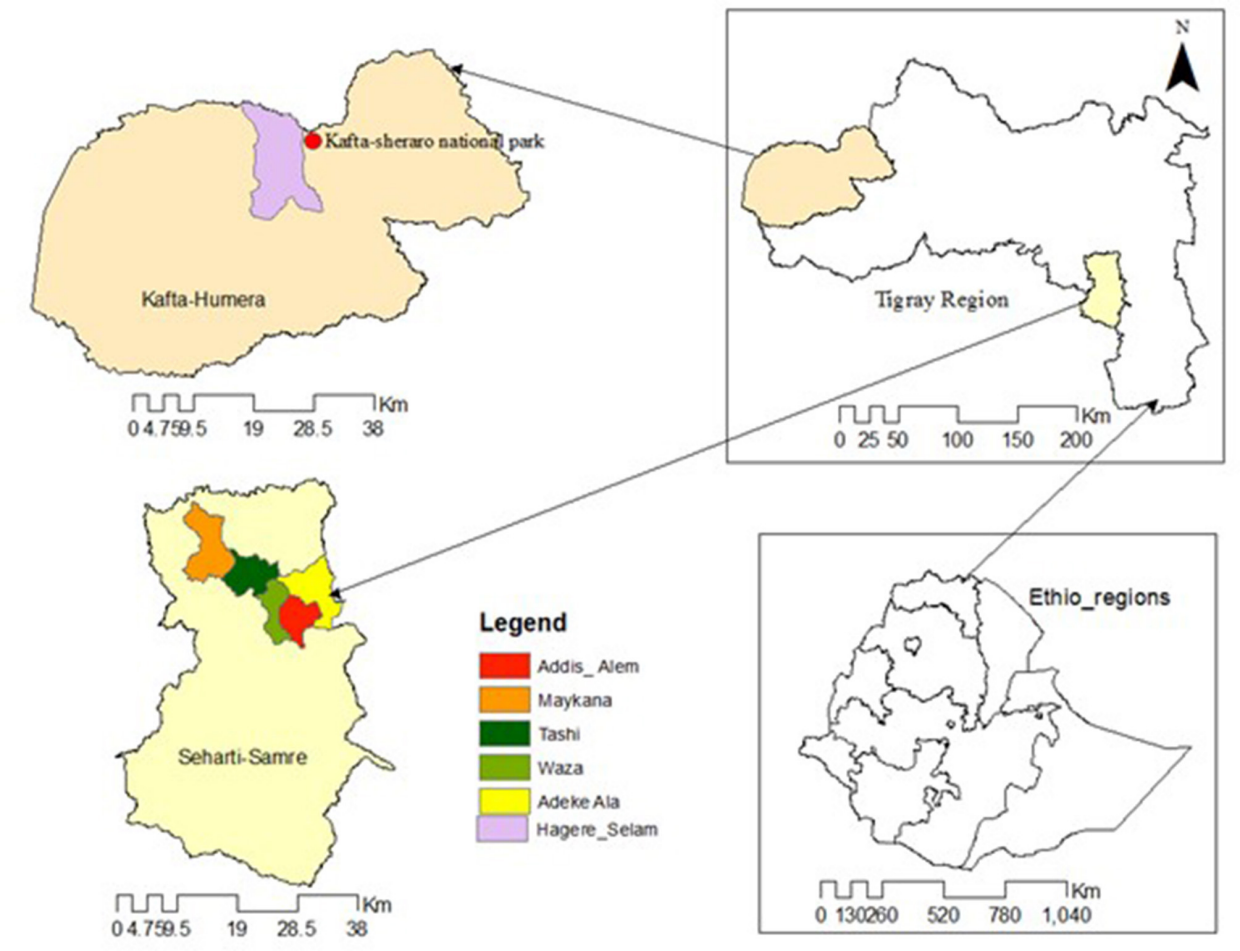
An administrative map of Kafta Humera and Seharti Samre districts with the one and five investigated villages, respectively, of Tigray region during the FMD outbreak in 2019.

During late October 2019, FMD outbreaks in cattle were suspected in one village of Kafta Humera district, western zone of Tigray region (Figure 1). The FMD cases were later detected in Seharti Samre district in the southeastern zone of Tigray region, where it massively affected the cattle population from the end of November 2019 to mid-December 2019. This study was conducted to (a) describe the history of the 2019 FMD outbreak in Tigray region, (b) investigate the causing FMDV serotype of the outbreak, and (c) highlight the potential source for the transmission of FMD to the cattle population in Kafta Humera district.

## Methods

### Study area

The study was conducted in the selected villages of Kafta Humera and Seharti Samre districts of the western and southeastern zones of Tigray region, respectively (Figure 1). Kafta Humera district was selected because it is the district in which the FMD outbreak was first reported in October 2019, while, later in the year, FMD cases were detected in Seharti Samre district.

Kafta Humera district borders to Eritrea to the north and Sudan to the west. It is located geographically between 36°25′ and 37°30′ longitude east and 13°30′ and 14°28′ latitude north, with a total area of 13,117 km^2^ (27). Begait cattle, which are indigenous and well known for their milk production potential, are the dominant breed (28). The cattle population was counted at 237,307 animals in 2019 (29). In Kafta Humera, free grazing pasturing of cattle is practiced during the wet season, while crop (predominantly sorghum, sesame, and cotton residues) is fed to cattle during the dry season. In Kafta Humera district, one village, Hagere Selam, was visited at the beginning of November 2019. The cattle population of Hagere Selam was counted at 9,811 individuals (29). Hagere Selam was the only village that was affected in this district, as reported along the Ethiopian surveillance system (from the local to the regional veterinary office). The village shares a large part of its border with the Kafta Sheraro National Park.

Seharti Samre district is located between 12°30′ and 13°29′ latitude north and 38°59′ and 39°26′ longitude east in Southeast Tigray, covering 2,724 km^2^. The district hosts a cattle population of 123,466 animals in 2019 (30). The farming system is traditional with mixed crop-livestock system and characterized by limited pasturing and feeding of crop residues predominantly from barley and wheat. The predominant indigenous cattle breeds are locally named Arado, which have the ability to resist the period of drought. Five villages were visited from the end of November 2019 to mid-December 2019. These villages were the only affected areas in the outbreak within the district, according to the district animal health coordinator.

### Data collection

#### Collection of case data and group discussions

In Kafta Humera and Seharti Samre districts, the affected villages, the number of cattle at risk, and the number of disease and mortality cases were received from the district animal health (AH) coordinator. The AH coordinator is a veterinarian who coordinates veterinary services in the district. Field veterinarians are able to notice the cattle with FMD that show typical clinical signs (ulcerative vesicular lesions in the mouth and legs and inter-digital cleft) together with the clear seasonal pattern in which the outbreaks occur.

In addition, a group discussion was held in Hagere Selam on 9 November 2019 using a participatory approach to investigate the factors that may have caused or are associated with the FMD outbreak. The group discussion was led by the investigating team of two researchers and one veterinary regional laboratory expert and three local experts (government employed graduate veterinarians), and 13 farmers from Hagere Selam village participated. The farmers were randomly selected from the sample frame of all farmers in the village. First, each farmer responded to a pre-defined questionnaire individually in an interview. The questionnaire was designed and applied in local language Tigrigna and translated into English for the analysis (**Table 3**). In addition, nine questions were asked on the farming system, grazing pattern, watering system, animal housing system, geographical location, intensity of wildlife and domestic animal interaction (based on farmers' observation), and history of FMD vaccination. Second, information collected in the interviews was discussed in a group discussion with the three local experts in a way that the discussion and criticism were made within the group, and then, the most shared idea was recorded as the final consensus. Data related to the total number of cattle per farm and the number of infected cows, oxen, and calves during the FMD outbreak were received from the district veterinarian coordinator, after consulting with the village animal health technicians.

No group discussion was performed in the Seharti Samre district because of the limited resources allocated to this district as the government gives attention to the outbreak response. Data related to farming system, grazing type, water source, housing system, wildlife interaction, and treatment of sick animals for this district were reported during sample collection by the district veterinarian coordinator.

#### Biological sample collection

To confirm the suspected clinical signs caused by FMD, feet epithelial tissues of active aphthous ulcer were collected from 22 cattle with clinical symptoms, 10 samples from Kafta Humera (from animals of farmers participated in the group discussion), and 12 samples from Seharti Samre district, collected by regional veterinary laboratory experts. Animals were sampled at their home pen, and the samples from cattle of the same pen were pooled within the same test tube containing virus transport medium, according to the WOAH guideline (31). This led to five and seven test tubes in Kafta Humera and Seharti Samre districts, respectively. The samples were collected from fresh lesions of cattle, showing symptoms, using a surgical blade. To avoid transmission of FMD during the sample collection, bio-security measures (glove and disinfectants) were applied and changed between each household visit. After collection, the samples were shipped to Tigray Veterinary Regional Laboratory on 12 November 2019 and 29 November 2019 from Kafta Humera and Seharti Samre districts, respectively, by maintaining the cold chain (+4°C) during the transport. Upon reception of all samples at the Tigray Veterinary Regional Laboratory, which is a temporary storage, the samples were immediately forwarded to the Animal Health Institute (AHI) in Addis Ababa for laboratory analysis.

### Laboratory analysis

Laboratory investigation was applied for the detection and typing of FMD viruses of serotypes O, A, C, Asia1, SAT1, and SAT2 in homogenates of epithelium vesicles and vesicles fluid. The sandwich enzyme-linked immunosorbent assay (ELISA) (lot No. 01-2018 180122a, Pirbright Institute, UK at AHI, Addis Ababa, Ethiopia) was applied for FMDV antigen detection and serotyping, with selected combinations of anti-FMDV coated and conjugated monoclonal antibodies (MAbs).

### Data analysis

The recorded data on the cattle population and cases showing clinical FMD symptoms were stored in Excel. Excel was used for descriptive statistics, including the distribution of sex (cows vs. oxen) and age (calves defined as cattle younger than 2 years of age vs. adult cattle older than 2 years of age) classes within the population at risk and the calculation of the morbidity proportions. Morbidity proportions were calculated by dividing the number of diseased animals by the respective population at risk. The chi-squared test was used to compare population structure between districts and morbidity proportions across age and sex distributions and between districts using the packages *PropCIs* and *stats* of R statistical software (https://cran.r-project.org/). Association between housing system, water source, type of treatment, and intensity of wildlife–cattle interaction (number of wildlife animals observed around dwellings and grazing pastures) with the morbidity proportion was assessed for the 13 farms in Hagere Selam for which the farmers were interviewed by applying univariate linear models in R software (the *stats* package), and a *p*-value of 0.05 was considered significant. The same model was used to quantify the association between cattle density and morbidity proportion in the five villages of Seharti Samre. The analysis of the qualitative data collected during the interviews and group discussions was purely descriptive.

## Results

### Description of the outbreak

The demography of the studied cattle population between the two districts differed significantly in sex (*p* < 0.001) but not in age (*p* = 0.435) (Tables 1, 2). The proportion of female cattle dominates with 91.8% in Kafta Humera district, due to the market-oriented use of cattle for milk production and calves replacement. Bulls and heifer are often sold rapidly. The use of Begait cattle for draft power is very low. In contrast, the proportion of males (50.9%) is slightly higher than females (49.1%) in Seharti Samre since the main purpose of keeping cattle is for draft power. The proportions of calves are 10.6 and 10.2%, comparable between the two districts.

**Table 1:** Proportion of FMD clinical cases observed during the outbreak investigation in cattle across age in the studied villages of Kafta Humera and Seharti Samre districts, Ethiopia, in 2019.

**Study districts**	**Village name**	**Total pop**	**Village Area (km^2^)**	**Pop. density**	**Calves** ^**a**^		**Cow/heifer**		**Oxen** ^**b**^		**Total No. inf. (%)**
					**Total no**.	**No. inf. (%)**	**Total No**.	**No. inf. (%)**	**Total No**.	**No. inf. (%)**	
Kafta Humera	Hagere Selam	9,811	16.37	599	1,037	165 (15.9)	8,336	4,012 (48.1)	438	122 (27.8)	4,299 (43.8)
Seharti-Samre	Addis Alem	3,355	32.11	104	341	303 (88.8)	1,633	1,454 (89)	1,381	1,229 (88.9)	2,986 (89)
	Adi-kala	4,589	53.04	86	439	307 (69.9)	2,132	1,512 (70.9)	2,018	1,439 (71.3)	3,258 (70.9)
	Tashi	4,141	105.3	39	336	288 (85.7)	2,341	2,013 (85.9)	1,464	1,259 (85.9)	3,560 (85.9)
	Waza	1,704	67.24	25	325	295 (90.7)	533	485 (90.9)	846	772 (91.2)	1,552 (91)
	Maay- Kana	3,132	124.5	25	287	205 (71.4)	993	710 (71.5)	1,852	1,383 (74.7)	2,298 (73.3)
	**Sub-total**	16,921	382.19	44	1,728	1,398 (80.9)	7,632	6,174 (80.8)	7,561	6,082 (80.4)	13,654 (80.6)
**Total (both districts)**	26,732									17,953 (67.2)

Pop, population in the village; inf, infected animals.

^a^Calves are defined as cattle younger than 2 years of age.

^b^Oxen are defined as male cattle that are castrated.

Population density was estimated by the number of cattle in the villages over the total area coverage (km^2^) of each village.

**Table 2:** Proportion of FMD clinical cases observed during the outbreak investigation in cattle across sex in the studies villages of Kafta Humera and Seharti Samre districts, Ethiopia, in 2019.

**Study districts**	**Village name**	**Total pop**	**Sex**			
			**Male**	**No. inf. (%)**	**Female**	**No. inf. (%)**
Kafta Humera	Hagere selam	9,811	801	193 (24)	9,010	4,106 (45.6)
Seharti Samre	Addis Alem	3,355	1,562	1,389 (88.9)	1,793	1,597 (89)
	Adi-kala	4,589	2,298	1,628 (70.8)	2,291	1,630 (71.1)
	Tashi	4,141	1,652	1,409 (85.3)	2,489	2,151 (86.4)
	Waza	1,704	1,067	964 (90.3)	637	588 (92.3)
	Maay-kana	3,132	2,037	1,529 (75.1)	1,095	769 (70.2)
	Total	16,921	8,616	6,919 (80.3)	8,305	6,735 (81.1)

Pop, population; inf, infected animals.

In Kafta Humera, the investigated FMD outbreak was reported to have started at the end of October 2019 according to the observations of the farmers and experts involved in this study. The total FMD morbidity proportion in the examined villages was 4,299/9,811 (43.8%). Morbidity proportion significantly differed between age classes (*p* < 0.001), with the highest found in adult cows and heifers (48.1%), followed by 27.8% in oxen and 15.9% in calves (Table 1). The morbidity proportions were found to be significantly different (*p* < 0.001) between male and female cattle with 24 and 45.6%, respectively (Table 2).

In Seharti Samre, the outbreak commenced at the end of November 2019 as reported from the district veterinarians. The overall morbidity proportion reported in the visited villages at the end of the outbreak was 13,654/16,921 (80.6%). The morbidity proportions significantly differed between the villages (*p* < 0.001), ranging from 70.9% in Adi-kala to 91% in Waza (Table 1). The morbidity proportions of FMD in Seharti Samre did not differ significantly between age groups (*p* = 0.754) with similar values of ~81% for each category. In addition, the morbidity proportions did not differ significantly between males with 81.1% and females with 80.3% (*p* = 0.199). We have followed the outbreak development until the end through telephone conversation with the district veterinarians (our enumerators). However, no mortality was reported among FMD suspected cattle in both districts.

Our results revealed that none of the risk factors, housing system, water source, intensity of interaction with wildlife, and type of treatment used were significantly associated with the morbidity proportion of the cattle of interviewed farmers (*p* > 0.48). The association between the population density and morbidity proportion for the five villages of Seharti Samre was also statistically non-significant (*p* = 0.93).

No clinical signs of FMD in other susceptible domestic animal species (sheep and goat) were detected throughout the outbreak in both districts.

### Clinical signs and symptoms observed

**Figure 2:**
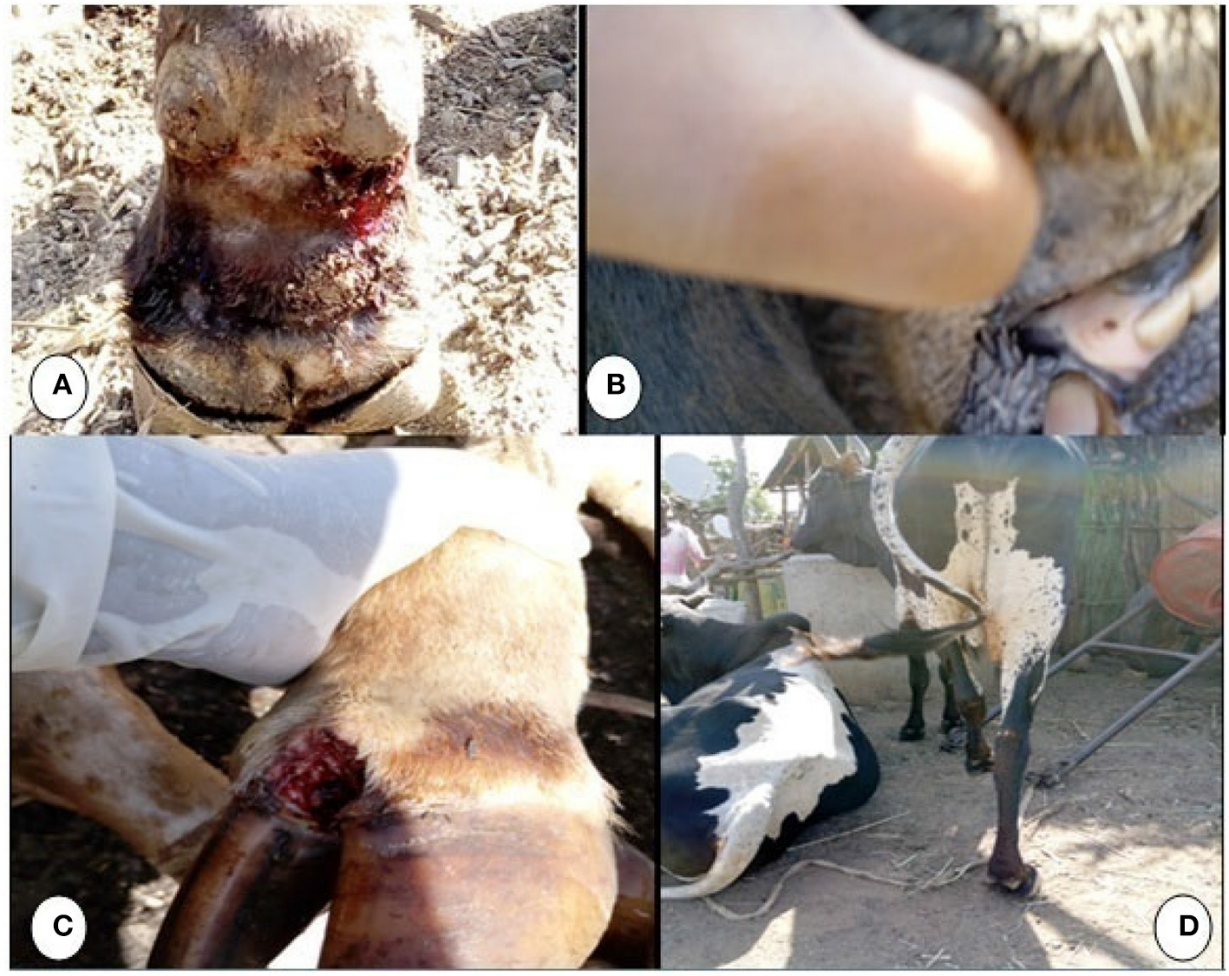
Clinical lesions of FMD during an FMD outbreak in Kafta Humera and Seharti Samre districts, Ethiopia, in 2019: **(A)** hoof sloughing with maggots; **(B)** spot lesion on palate; **(C)** abrasive lesion of inter-digital space; **(D)** lameness.

The clinical signs observed by the experts and farmers were typical to FMD: pyrexia up to 40°C, vesicles on the inter-digital cleft with or without fly maggots leading to lameness, and lesions on the oral cavity, hard palate, and dental pads, resulting in drooling or hyper-salivation (Figure 2). Similar clinical signs were observed in both districts.

### Laboratory diagnostics

Out of the five pools collected in Kafta Humera, three were found positive for serotype O (60%); one pool was found positive for FMDV but with unidentified serotype and one pool was found negative for FMDV. Out of seven pools of samples collected in Seharti Samre district, two were found positive for FMDV serotype A (28.6%) and the remaining five pools were tested negative for FMDV. According to AHI, the sensitivity and specificity of this test are above 95%, though cross-reactivity between serotypes A and O is observed.

### Participatory interviews and group discussions

Outcomes of the interviews and group discussion from Kafta Humera district revealed that a mixed crop-livestock farming system is applied in all farms (Table 3). Natural pasturing is the most important feed source used for cattle during all seasons of the year. Most farmers explained that the grazing land is entirely communal. In addition, the conservation of sorghum residues, sesame residues, and hay are commonly supplemented feed during the dry season. Supplementation with commercial feeds has never been reported. The main sources of water were described to be rivers (61.5%) and bore wells (38.5%). Nine farmers explained that the housing system is usually open fenced (simple fences made of wood and not roofed; 69.2%) and far from human dwellings. Most often, cattle move from human dwellings to an open pasture in June when the rain starts and move back to dwellings during October to graze on crop-aftermaths. These green grass pastures used during the rainy season in Kafta Humera district are located close to the national park. The majority of respondents stated that the interaction with wildlife happens often and throughout the year. This is due to the closeness of cattle pens to the Kafta Sheraro National Park (~5 km). Grazing cattle in the park is prohibited. Park guardians protect livestock from the entrance to the national park, and punishment for farmers not following the regulation is considerable. However, no physical fence between the livestock grazing land and the national parks is established. In October 2019, FMD typical clinical signs were reported by the farmers in nine wild deer, in which three mortality cases were observed. After a few days, FMD clinical signs were also detected in cattle of the nearby village, Hagere Selam (Personal information, Adehanom Baraki).

**Table 3:** Group discussion outcomes from 13 farmers supported by three local experts during FMD outbreak investigation in Kafta Humera district in November 2019 and Seharti Samre reports on husbandry practices and other parameters.

**Type of questions**	**Kafta Humera (Hagere Selam village)**		**Seharti Samre (villages AdisAlem, Adi-kala, Tashi, Waza, and Maay-kana)**
	**Category**	**Response**	
What kind of farming system do you follow?	Mixed crop-livestock farming system	13	Mixed crop-livestock farming system
What kind of grazing system do you follow?	Entirely communal during the summer season (rainy) plus supplementary feed provided during the dry season (residues of sorghum, sesame, and hay)	13	mostly at the home relay on crop residue products except for limited seasonal pasture in Adis-Alem
What is the main water source?	Pipe water	0	River in all except borehole in Adis-Alem
	River	8	
	Bore hole	5	
What is the main housing system?	Stall barn	0	All fenced and roofed
	Fenced and roofed	4	
	Open fenced, not roofed	9	
Is wildlife and domestic animal interaction observed in your area?	Yes	13	No wildlife observation
	No	0	
If yes, how often are contacts observed?	All times	10	
	Sometimes seasonal	3	
What treatment do you use in case of FMD sick animals?	Traditional	8	Modern and Traditional in all villages
	Modern	5	
List of traditional treatments	Honey, Iodine salt		
List of modern treatments	Iodine, Savlon, wound spray, antibiotics		
Was FMD vaccination service performed in 2019?	No	13	No
When does FMD usually appear?	Most often at the end of the harvesting season (January)	13	Most often at the end of the harvesting season (January)

Eight farmers used traditional treatment (61.5%) to treat FMD clinical lesions in their cattle. This includes adding honey or iodinated salt to the lesions. Five farmers used modern treatment (38.5%), such as antibiotic injection, wound spray, application of iodine tincture, and Savlon wash. All farmers stated that FMD most often occurs at the end of the harvesting season, which is around January. The farmers reported that there was no FMD vaccination campaign performed in the study area during the entire outbreak year (2019).

In Seharti Samre district, as per the expert report, the selected five villages entirely follow a mixed crop-livestock farming system. The grazing system is mostly home-based with feeds on crop residues, except during the harvesting season (feed on aftermaths) and seasonally limited pasturing (mostly at Maay-kana village). The river is the most often reported (80%) water source, while the housing system in all villages is based on fenced and roofed structures. No FMD susceptible wildlife species were observed in the five villages of Seharti Samre. Regarding the treatment options, traditional and modern treatment methods were used in all villages (Table 3).

## Discussion

According to the case reports, 43.8% of the cattle population in Kafta Humera and 80.6% in Seharti Samre were infected with FMDV, resulting in a massive continuous epidemic of FMD at the end of 2019 in the two studied districts. The large difference of the morbidity between the two districts is difficult to explain but may be because Seharti Samre was visited the latest. In addition, farmers in Kafta Humera are more aware and the way they try to contain the disease was extraordinary (from traditional to modern treatments). Outbreaks of similar sizes were reported in different parts of Ethiopia during the previous years, with an increasing size and frequency of outbreak occurrence of FMD in cattle from year to year (10, 13, 15, 32, 33).

An overall high morbidity proportion was observed in the sampled villages throughout the outbreak. All age groups were affected, including adult cows and oxen. This can result in a high reduction in milk production and shortage of draft power for the farmers (17, 34). By considering the importance of Begait cattle breeds, which are known for their dairy potential (28, 35), the disease can result in huge milk loss and a devastating economical loss for the region in various agricultural sectors (34, 36). Estimation on economic loss would be a relevant additional analysis for future studies.

Our results show that adults are more affected with FMD than younger animals in Kafta Humera districts. Similar reports have been observed in Gamo Zone, southern Ethiopia (37). This can be due to calves being protected by passive maternal immunity and age-related physiological factors (37), and calves in this area have limited access to grazing until the age of 2 years. We also found that, in Kafta Humera, females were more affected than males, an observation that was not reported in the Seharti Samre district. In the study conducted in the Gamo Zone, there was no difference reported in morbidity proportion between males and females (37). It is noteworthy that there might be several factors not captured by this study that may explain our findings of female and adult animals being more infected, and therefore, these results should be treated with caution.

No mortality was observed in the diseased cattle, which is similar to the finding in a study conducted in Bangladesh (38). This might be due to certain immunity developed after continuous exposure to the natural infection of FMDV, which is common in endemic areas. In addition, no sheep and goat were reported to be infected during the FMD outbreak. This could be due to the fact that the clinical signs are much more difficult to detect than cattle.

The majority (7/12) of pools from the 22 samples collected were negative for FMDV by ELISA. Although some lesions may have been wrongly identified as suspected FMD by farmers, they are typically correctly identified as FMD lesions by experienced veterinarians. More likely, the negative results might be due to improper sample transportation (failure of maintaining cold chain) because the laboratory (AHI) in Addis Ababa is more than 1,000 km away from the outbreak site. Investment into high-quality sample transportation tools (for example, improvement in cold chain or reagents allowing non-cooled transports) is therefore crucial. In addition, decentralization of laboratories would substantially reduce the transport distance required and would allow more timely analysis of the samples. In FMD endemic developing countries, the underlined detrimental factors can compromise the ability to detect and characterize FMD virus properly (39). Nevertheless, the main aim of the laboratory analysis was to confirm the presence of FMDV and to identify the circulating serotypes. Since the FMDV serotypes identified in the two districts are different (serotype O in one district and serotype A in the other district), we hypothesize that the two outbreaks evolved independently from each other. Although we cannot rule out one single outbreak source with both serotypes (considering the small sample size of laboratory testing), the identification of two distinct serotypes points out the high risk of FMD incursion and the relevance of identifying involved FMDV serotype to target disease control measures, e.g., by vaccination. Serotypes O and A are among the frequently reported FMDV serotypes in Ethiopia (40, 41). Owing to the very small sample size, there is a chance that other serotypes were missed during the outbreak investigation.

The discussion on possible sources for the FMD outbreak in Kafta Humera by the group discussion revealed that FMD typically appears at the end of the harvesting season when all cattle of the surrounding villages graze freely in the pasture lands and fields. The harvesting season usually starts in October and ends in January. This observation by the farmers is in line with investigations of FMD outbreaks in Ethiopia between 2009 and 2018 that also revealed the high-risk period for outbreaks between December and January (10). This was also observed for the reported outbreaks here in 2019, although the outbreak started earlier in the middle of the harvesting season. In Hagere Selam village, cattle share common grazing land and natural watering points not only with domestic animals of neighboring villages but also with wildlife, increasing the chances of disease transmission between domestic and wild animals. Approximately 42 wildlife species are living in the Kafta Sheraro National Park, including potentially FMD susceptible species such as greater kudu (*Tragelaphus strepsiceros*), bush duiker (*Sylvicapra grimmia*), defasa waterbuck (*Kobus ellipsiprymnus*), roan antelope (*Hippotragus equines*), red-fronted gazelle (*Eudorcas rufifrons*), and wild deer (*Odocoileus virginiana*) (42). Many farmers reported that many of the above species of wildlife were observed daily in the surrounding grazing and watering points. Common grazing and watering of domestic and wild animals have been observed in the reported outbreak here, although we did not find any association between the intensity of observed contacts and the morbidity proportion on the farm level. The risk for FMD transmission between wildlife and domestic animals was discussed in many other studies in Africa (3, 10, 13, 15, 32, 43, 44).

Another potential source of FMDV infection is cross-bordering transmission when local traders illegally exchange cattle from Sudanese and Eritrean traders. The Kafta Sheraro National Park also borders to Eritrea, and transmission of diseases from and to Eritrea to the national park might be crucial. Outside Ethiopia, the Begait cattle breed (Barka in Eritrea) is kept mainly by a small ethnic group Beni-Amer, who resides in Eritrea and Sudan. These farmers are specialized in breeding management (selecting and keeping best Begait breeds). Before the war of Ethio-Eritrea has started on 1998, they also lived in Kafta Humera districts. As a result, cattle keepers in Kafta Humera district believe that Begait breeds kept by “Beni-Amer” farmers have a better reproductive performance. Thus, traders from Tigray, particularly in Kafta Humera district, buy bulls and heifers illegally from Sudan and Eritrea. In Seharti Samre, the source of the FMD outbreak in 2019 is more uncertain. In addition, here, illegal trade with Eritreans via Sudan at the closed Ethio-Eritrea border is a potential source of infection.

In summary, the potential source of outbreak is not clear in both districts. Sahle et al. (32) suggested that uncontrolled cross-border animal movement to neighboring countries, such as in southern Ethiopia from the Borana pastoral area to Kenya and vice versa, make Ethiopia the only country in Africa with circulating five serotypes of FMD virus (O, A, C, SAT1, and SAT2) (18). Studies investigating FMD serotypes in wild ruminants of the Kafta Sheraro National Park are lacking but should be promoted to generate evidence of potential source of FMD in this region. According to Auty et al. (45), FMD outbreaks associated with activities such as hunting, shooting, stalking, and equestrian events in national parks were described as significant.

Due to the highly infectious nature of FMDV, FMD outbreaks should be addressed at an early stage with effective control measures to prevent disease propagation (46). Vaccination against FMDV serotypes that are circulating in the area is an effective tool to combat and prevent outbreaks in endemic areas (47). Prevention of outbreaks through regular vaccination is practiced in many FMD-endemic countries, including Ethiopia. This also applies to the Tigray region, where vaccination was provided at a regular basis prior to 2019. However, due to the high costs of FMD vaccination compared with other vaccines, mainly commercialized farms are vaccinated on a yearly basis, whereas small holdings in Tigray region often do not afford vaccination. In the current outbreak, farmers stated that no vaccination against FMD was given during the year 2019 due to political unrests, which increases the risk for the onset of an outbreak. In addition to the lack of annual vaccination campaigns, usage of vaccines not adapted to the circulating serotypes is also problematic. In the outbreak investigation described here, laboratory analyses detected that three of the pooled tissue samples were tested positive for FMDV serotype O and two samples were tested positive for serotype A. Previous outbreak reports identified the circulating serotypes O, A, C, SAT 1, and SAT 2 in Tigray (12, 13), with serotypes O being more prevalent in the previous years (13). To achieve effective vaccination, it is required to know the circulating serotype because cross-immunity of vaccines between serotypes is weak (22). In Ethiopia, the current circulating FMDV serotypes are not frequently studied. As a result, the existing vaccines (covering serotypes O, A, and SAT 2) may not provide the optimal protection.

### Limitations

The limitations of this study were that no focus group discussion was conducted in Seharti Samre, and that a limited number of farmers and animal health personnel were available to collect morbidity data from thousands of animals. This could influence the morbidity proportions during the current outbreaks. There is some evidence that the sources of infection in Kafta Humera are wildlife; however, this is speculative and transmission could have also occurred from livestock to wildlife (48). In addition, we did not collect information on the source of the outbreak in Seharti Samre. Moreover, most FMD suspected cases by clinical signs have not been laboratory confirmed. However, since veterinarians are experienced with this disease and FMDV has been identified in selected samples, there is a high chance that cattle identified as suspected were FMD positive.

## Conclusion

The current outbreaks of FMD in two districts of the Tigray region in Ethiopia led to massive morbidity in the cattle population. The lack of regular vaccination and lack of knowledge about the circulating serotypes before administration of vaccines are among the reasons why FMD outbreaks reoccur in a regular pattern in Tigray. Wildlife may serve as a reservoir of FMDV and disease transmission to cattle population in the Kafta Humera district. Therefore, the following recommendations should be considered:

- Further investigation regarding the (molecular) epidemiology of circulating FMDV serotypes in the wildlife and domestic population should be performed, with the aim to confirm causality, confirm the source of FMD transmission, and identify the reservoir population.
- Regular vaccination should be practiced for the identified circulating serotypes in the Tigray region with sufficient coverage.
- Unauthorized animal movement from and to the national park should be strictly controlled, and livestock movements to national parks should be reduced by guardians and fences where required.
- Improving the laboratory capacity (procurement of reagents, transport media to ensure cold chain, PCR material, and serological diagnostic test kits) of the Tigray regional veterinary laboratory is immeasurably helpful for early disease detection and rapid outbreak responses.

## Data availability statement

The original contributions presented in the study are included in the article/Supplementary material, further inquiries can be directed to the corresponding authors.

## Ethics statement

All study protocols for animal and human were approved by Mekelle University College of Veterinary Science Animal Ethics Committee (AEEC) and all the methods were conducted according to the Guidelines on Care and Use of Animals for Scientific Purposes available in www.mu.edu.et. Before conducting the interview, the objectives, expected results and benefits of the study were explained to got owners and informed consent to participate was obtained from all participant farmers. The patients/participants provided their written informed consent to participate in this study. Written informed consent was obtained from the owners for the participation of their animals in this study.

## Author contributions

GA and GG prepared the outbreak template and coordinated the overall outbreak investigation team. AT, LS, NG, and BA collected data and tissue samples. AT and LS lead the outbreak investigation in the field. AT and BA followed laboratory analysis and collected laboratory results. GA compiled and conducted descriptive analysis of the data. SD analyzed the data statistically in R software for association of dependent and independent variables. AT conceptualized and drafted the manuscript. GA and SD edited and reviewed the drafted manuscript. AT, GA, and SD prepared, read, commented, and approved the final manuscript for publication. All authors contributed to the article and approved the submitted version.
